# Using Pareto optimality to explore the topology and dynamics of the human connectome

**DOI:** 10.1098/rstb.2013.0530

**Published:** 2014-10-05

**Authors:** Andrea Avena-Koenigsberger, Joaquín Goñi, Richard F. Betzel, Martijn P. van den Heuvel, Alessandra Griffa, Patric Hagmann, Jean-Philippe Thiran, Olaf Sporns

**Affiliations:** 1 Department of Psychological and Brain Sciences, Indiana University, Bloomington, IN, USA; 2 Rudolf Magnus Institute, Utrecht, The Netherlands; 3 Signal Processing Laboratory (LTS5), Ecole Polytechnique Fédérale de Lausanne (EPFL), Lausanne, Switzerland; 4 Department of Radiology, Lausanne University Hospital (CHUV) and University of Lausanne (UNIL), Lausanne, Switzerland

**Keywords:** graph theory, network science, brain connectivity, diffusion imaging

## Abstract

Graph theory has provided a key mathematical framework to analyse the architecture of human brain networks. This architecture embodies an inherently complex relationship between connection topology, the spatial arrangement of network elements, and the resulting network cost and functional performance. An exploration of these interacting factors and driving forces may reveal salient network features that are critically important for shaping and constraining the brain's topological organization and its evolvability. Several studies have pointed to an economic balance between network cost and network efficiency with networks organized in an ‘economical’ small-world favouring high communication efficiency at a low wiring cost. In this study, we define and explore a network morphospace in order to characterize different aspects of communication efficiency in human brain networks. Using a multi-objective evolutionary approach that approximates a Pareto-optimal set within the morphospace, we investigate the capacity of anatomical brain networks to evolve towards topologies that exhibit optimal information processing features while preserving network cost. This approach allows us to investigate network topologies that emerge under specific selection pressures, thus providing some insight into the selectional forces that may have shaped the network architecture of existing human brains.

## 1. Introduction

The emergence of network science and the increasing availability of brain connectivity data have recently opened up a network-based perspective on brain function. Studies in this area use diverse mathematical and computational tools to study the architecture of brain networks and its role in the dynamics of information processing [1,2]. In the human brain, the use of diffusion imaging techniques to detect white matter pathways connecting anatomical brain regions has enabled the mapping and analysis of structural brain networks. While descriptive studies have identified a number of characteristic topological attributes [3–5], the fundamental selectional forces and factors that have shaped human brain network topology remain poorly understood. Three candidate factors explored here are network cost [6–8], network communication efficiency [9–11] and dynamic complexity [12–14].

It has been recognized for over a century that one fundamental factor shaping neuronal morphology and connectivity is that brain networks are embedded in space. A major consequence of spatial embedding is that the generation, maintenance and use of connections incur a cost, as connectivity consumes various resources such as wiring length [6–8] and metabolic energy [15]. Like in any biological system, these resources are limited and the need to conserve such resources places strong constraints on the topology of the system. Underscoring the importance of spatial embedding [16,17], many studies have shown that the topology of connections between individual neurons is strongly influenced by the spatial distance between them [18] and that cortical regions that are spatially close have high probability of being connected to each other [19–21].

However, spatial distance alone is insufficient to fully explain the connectivity patterns observed in brain networks. Structural brain networks are characterized by the existence of specific long-range pathways [22], highly connected regions (hubs) [23] and community structure [24,25], features that violate the concept of wiring minimization. In general, these connectional attributes are found in systems that achieve highly efficient global communication and whose components are highly clustered, e.g. small-world networks [26]. Many studies have shown that human brain networks are organized in an economical small-world manner, which tends to minimize wiring costs while supporting a few long-range connections that are thought to ensure high efficiency in global communication [22,27,28].

Another aspect of network organization relates to the brain's capacity to support a great diversity of dynamic patterns which are highly complex and essential to sustain a large number of competing functional demands. This diversity of dynamic patterns has been conceptualized as a ‘functional repertoire’ of network states that enables flexibility across a broad range of cognitive functions [29]. From a network perspective, this aspect points to the importance of local or specialized information processing in the cortex as well as the integration of information between different specialized regions [12,13]. It has been suggested that both aspects of information processing, integration and segregation, underlie the complex dynamics taking place in the network and that the patterns of structural connectivity found in the brain promote such complex dynamics [1,12,30].

None of these three factors alone is sufficient to account for all aspects of human brain network architecture. Instead, this architecture appears to represent a trade-off between these (and possibly other) competing factors, enabling economic information processing within a small-world topology [31]. Here, we explore the extent to which the structure of the human cortical brain network is optimally organized in order to achieve efficient and economical information processing. By defining a network morphospace and a multi-objective evolutionary algorithm that operates on the morphospace, we investigated the capacity of brain networks to evolve towards distinct biologically feasible topologies. This approach allows us to address important questions about what topological features emerge as a result of applying different types of selection pressure on the evolution of brain networks. For instance, how different are the networks selected for efficient information processing from networks selecting for certain dynamical properties? Furthermore, the analysis of a brain network morphospace aids in the understanding of actual brain network structure and provides a framework to study the structural variations to which brain networks are subject, due to individual differences or neurological degeneration.

Our analysis was performed over three different structural brain networks, obtained independently and from different imaging techniques. The analysis proceeds in three steps. First, we study the behaviour of our measures of information processing and dynamical complexity as the empirical networks are rewired towards two null models, namely, randomized and latticized networks. Both null models preserve crucial features of the corresponding empirical brain networks, such as network size, density, degree sequence and wiring cost. Second, we create a set of proximal networks by minimally perturbing the structure of the empirical networks. These proximal networks allow us to (i) quantify the sensitivity of our measures to small structural perturbations and (ii) depict the distribution of the proximal morphospace. Third, we use a multi-objective evolutionary algorithm to do a local exploration of the efficiency-complexity morphospace of brain-like networks. The aim of a local exploration is to investigate alternative biologically feasible topologies for structural brain networks. Through this kind of analysis, we are able to portray how and how much a sub-region of the morphospace (the region surrounding the empirical brain network) is filled; this in turn, can provide a picture of the underlying rules and constraints pervading the organization of brain networks.

## 2. Material and methods

### (a) Graph theory

To study brain connectivity, we apply methods from a branch of mathematics called graph theory [32–34]. In the context of graph theory, an anatomical brain network with *N* interconnected neural elements is modelled as a graph *G* = (*V*, *E*), where *V* is the set of vertices (or nodes) representing brain regions and *E* is the set of edges (links), representing white matter pathways. The size of a network is given by the number of nodes and connections composing the network, whereas the network density is defined as the number of existing connections divided by the maximum possible number of connections that the network can support. Formally, *G* is described by an *N* × *N* adjacency matrix *A*_G_ = {*a_ij_*}, where *a_ij_* = 1 if nodes *i* and *j* are connected and *a_ij_* = 0 otherwise. In addition, *G* is a weighted graph if there is a scalar associated with every connection, such that if *a_ij_* = 1, then there is a non-zero weight *w_ij_* assigned to the connection {*i*, *j*}. In the case of anatomical brain networks, we focus on two weighted matrices associated to the connections: a matrix of fibre densities *W*_G_ = {*w_ij_*} and a matrix of fibre lengths *L*_G_ = {*l_ij_*} estimated as in [3]. Thus, the degree of a node is given by the number of edges incident to the node, whereas the weighted degree is given by the sum of the fibre densities of the edges incident to the node. Finally, in this study, all the connections of anatomical brain networks are undirected, that is *a_ij_* = *a_ji_* for all pairs {*i*,*j*} and thus *A*_G_, *W*_G_ and *L*_G_ are all symmetric matrices.

### (b) Brain networks

Our analyses were carried out over three anatomical human brain networks (labelled LAU1, LAU2 and UTR; datasets are provided in the electronic supplementary material) constructed from data acquired independently in different imaging centres, using different acquisition protocols and different subject cohorts.

#### (i) LAU1

Five healthy right-handed male subjects (mean age 29.4 years, s.d. 3.4) were scanned on a 3-T Philips Achieva scanner. A high-resolution T1-weighted gradient echo sequence was acquired in a matrix of 512 × 512 × 128 voxels of isotropic 1 mm resolution. Diffusion spectrum imaging (DSI) was performed using a diffusion-weighted single-shot echoplanar imaging sequence (TR = 4200 ms; TE = 89 ms) encoding 129 diffusion directions over a hemisphere. The maximum diffusion gradient intensity was 80 mT m^−1^, the gradient duration was 32.5 ms and the diffusion time was 43.5 ms, yielding a maximal *b*-value of 9000 s mm^−2^. The acquisition matrix was 112 × 112, with an in-plane resolution of 2 × 2 mm. Following diffusion spectrum and T1-weighted MRI acquisitions, the segmented grey matter was partitioned into 998 regions of interest (ROIs). Following white matter tractography, connectivity was aggregated across all voxels within each of the 998 ROIs. Further details are available elsewhere [3].

#### (ii) LAU2

Forty healthy subjects (24 males and 16 females, 25.3 ± 4.9 years old) underwent an MRI session on a 3-T Siemens Trio scanner with a 32-channel head-coil. The magnetization-prepared rapid gradient-echo (MPRAGE) sequence was 1 mm in-plane resolution and 1.2 mm slice thickness. The DSI sequence included 128 diffusion-weighted volumes + 1 reference *b*_0 volume, maximum *b*-value of 8000 s mm^−2^ and 2.2 × 2.2 × 3.0 mm voxel size. The echo planar imaging (EPI) sequence was 3.3 mm in-plane resolution and 0.3 mm slice thickness with TR 1920 ms. DSI and MPRAGE data were processed using the Connectome Mapping Toolkit [35]. Segmentation of grey and white matter was based on MPRAGE volumes. Cerebral cortex was parcellated into 1000 equally sized ROIs [36] followed by whole-brain streamline tractography [37].

#### (iii) UTR

Imaging data were acquired from 25 subjects (17 males and eight females, 29.4 ± 7.7 years old). Diffusion-weighted imaging (DWI) was performed at 3 T, with two sets of 30 weighted diffusion scans (*b* = 1000 s mm^−2^), each set consisting of five unweighted B0 scans (*b* = 0 s mm^−2^) and 30 weighted scans (SENSE, p-reduction 3; gradient set of 30 weighting directions, TR = 7035 ms, TE = 68 ms, EPI factor 35; FOV 240 × 240 mm, 2 mm isotropic, 75 slices, second diffusion set acquired with a reversed k-space readout). Preprocessing of the DWI involved the following steps: (i) diffusion images were realigned, corrected for eddy currents and susceptibility distortions; (ii) diffusion profiles were fitted with a single tensor and deterministic streamline tractography was used to reconstruct streamlines; and (iii) streamlines were used to build subject-specific structural brain networks among 1170 equally sized randomly partitioned cortical parcels (nodes). For a detailed description see [38].

For all three datasets, all subsequent analyses and modelling were carried out on group consensus matrices, built by averaging over all existing connections (expressed as fibre densities) that were present in at least 25% of participants in each dataset. For this study, we limit the analysis to networks containing nodes and connections in the right hemisphere of the brain, for two reasons. First, inter-hemispheric connections are less reliably captured by diffusion imaging and more difficult to reconstruct with tractography [39]. Second, the computational cost of running the analyses and simulations proposed here in whole-brain networks was prohibitive.

### (c) Metrics of network performance

Complex network analysis has provided various metrics that aim to characterize different aspects of network topology [32,33]. Here, we selected four measures that jointly capture the performance of a network at combining integrated and segregated information processing in an economical manner.

#### (i) Wiring cost

This measure quantifies the cost of making and maintaining anatomical connections between neurons [8,15]. By assuming that the wiring cost is proportional to the wiring volume [28,31], we can express the cost of a single connection {*i*, *j*} as the product between its fibre density and length. Then, the total wiring cost of a network with *N* nodes is given by 
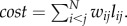


#### (ii) Efficiency of information processing

We approach the measurement of efficiency of information processing from the perspective of two different communication schemes, one based on the routing of information in a network, and the other one based on the diffusion of information within the network [11].

##### Routing efficiency

In this work, the measure *E*_glob_ defined in [9] is referred to as *E*_rout_ [11]. This measure is based on the shortest path length matrix *φ* = [*φ_ij_*] where the distance between a pair of nodes is computed in terms of the inverse of the fibre densities of the connections. Then, the routing efficiency is computed as follows:

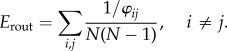



##### Diffusion efficiency

We start by defining a transition matrix *P* as the matrix whose elements *p_ij_* represent the probability of a random walker going from node *i* to node *j* in one step. If the transition probabilities are proportional to the fibre density of the connections, *p_ij_* = *w_ij_*/*k_i_*, where *k_i_* is the weighted degree of node *i*. Given a transition matrix, the mean first passage time (MFPT) between node *i* and *j* is defined as the average number of steps it takes a random walker starting at node *i*, to arrive at node *j* for the first time [40]. If the network is connected and has no self-connections, the MFPT between any pair of nodes is finite and can be computed as follows:

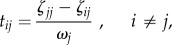

where the vector *ω* is the left eigenvector associated to the eigenvalue of value unity; *Z* = [*ζ_jj_*] is the fundamental matrix, computed as *Z* = (*I* − *P* + *W*)^−1^, where *I* is the *N* × *N* identity matrix, *P* is the transition matrix and *W* is an *N* × *N* matrix with each column being the vector *ω* such that 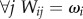. Diffusion efficiency is then defined as follows [11]:

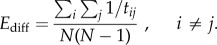



#### (iii) Neural complexity

Neural complexity (*C*_N_) is a measure that captures the coexistence of functional segregation and functional integration in a neural system [12]. *C*_N_ is a statistical measure of the dynamics of the system defined in terms of the mutual information between subsystems. *C*_N_ was originally defined in terms of the integration associated with a system of *n* neural components and a stationary stochastic process 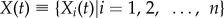, where *X_i_*(*t*) represents the activity of the *i*th neural component at time *t*. Integration is computed as 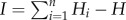, where *H* is the entropy of the entire system and *H_i_* is the entropy of the *i*th individual component. Then, neural complexity is defined as follows:

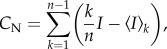

where 〈*·*〉_*k*_ denotes an average over all 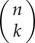 subsystems of size *k. C*_N_ tends to be low for systems whose components are either statistically independent or highly dependent; conversely, *C*_N_ is high for systems whose components are (on average) independent in small subsets and increasingly dependent in subsets of increasing size.

##### Computation of *C*_N_

Note that in the definition of *C*_N_ given above, the number of subsystems of size *k* = 1, 2,…, *n* − 1 increases exponentially with *n* as 2*^n^*. The subsequent combinatorial explosion makes it unfeasible to calculate *C*_N_ for large values of *n*. To overcome this problem, *C*_N_ can be approximated by a *sampling method*, which consists of taking *M* random samples of subsystems of size *k* (for *k* = 1,…, *n* − 1); as *M* increases, the approximation approaches the exact value of *C*_N_; hence, *M* must be sufficiently large to get an accurate approximation and this computation becomes very intensive. Alternatively, a computationally less intensive approximation for *C*_N_ has been proposed [41], used here to compute *C*_N_ values. This approximation assumes that the correlation between the activities of neural components is small and that the dynamics of the system are stable; *C*_N_ can then be approximated as

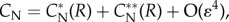

where

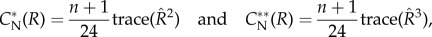

and 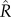 is the correlation matrix with elements equal to zero in the diagonal 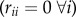.

To derive correlation matrices from the structural connectivity matrices, we implemented a linear model of neural activity described in [42]. This model is based on the linearization of a coupled neural system driven around a fixed point by spatially and temporally independent Gaussian noise sources. The correlation of the dynamics, matrix *A*, can be obtained analytically as 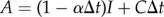. The coupling matrix *C* used here is the structural connectivity matrix, *I* is the identity matrix and *α* is the rate of activation leakage per node (here set to *α* = 2). Finally, the condition for weakly coupled neural elements is met when the spectral radius (the absolute value of the largest eigenvalue) of the covariance matrix is smaller than unity.

The condition of weakly coupled neural elements comes from the assumption that neural dynamics can be approximately characterized by a stationary multivariate Gaussian process. By implementing a Gaussian neural model as a linear process, the computation of *C*_N_ is remarkably simplified, given that it is possible to express the interactions between neural elements (i.e. entropies and mutual information) in terms of a covariance matrix that can be derived analytically from the network's connectivity matrix. However, the linearization of the neural model is an approximation in the weakly coupled near-linear regime of the nonlinear dynamics of the system. Linear approximations are commonly used in neuroscience to model large-scale neural systems [43], which is what the nodes of the networks used in this work represent.

### (d) Network morphospace

**Figure 1.**
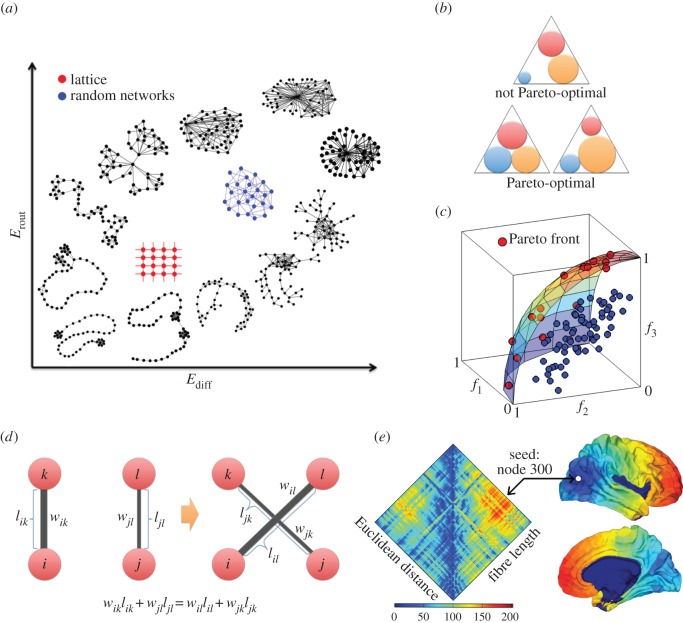
(*a*) Diagram of a communication-efficiency morphospace for toy-networks; the location of a toy-network within this morphospace of abstract networks can be associated with specific aspects of network structure that favour two distinct communication schemes: diffusion-based communication (*E*_diff_) and routing-based communication (*E*_rout_). (*b*) Geometric example of Pareto optimality: the area of three circles represents three objectives to be maximized; circles are constrained to be contained within an equilateral triangle and cannot overlap with each other. There are several solutions to the problem; the top triangle shows a solution that could be improved by increasing the area of the blue circle; thus, it is not Pareto-optimal. The two bottom triangles show solutions in which the area of none of the circles can be increased without having to decrease the area of another circle; therefore, the solutions are Pareto-optimal [45]. (*c*) Example of a Pareto front, where three objective functions are to be maximized. All the points in the plot represent feasible solutions; however, only the red points belong to the Pareto front. (*d*) Rewiring rule: the weights of the edges {*i*, *l*} and {*j*, *k*} are randomly selected, provided that the total wiring cost is preserved. (*e*) Matrix of Euclidean distances (left side) and interpolated fibre lengths (right side) between all pairs of nodes of the LAU1 dataset. The colour map on the human cortex images represents the fibre lengths after interpolation between node 300 (whose location is indicated with a white circle) and all other nodes of the LAU1 network.

The concept of morphospace originated in the context of evolutionary biology [44]. It provides a framework to map all the possible biological forms that can result by varying the parameter values of a geometrical or mathematical model of form. Most importantly, it allows the identification of forms that have been produced in nature and forms that have not. The parameters of a model of form define the axes or dimensions of a morphospace, where different locations within each dimension specify the parameter values and are associated with a particular biological form. Here, we extend the concept of morphospace to encompass network structure; hence, the dimensions of a network morphospace are given by network structural measures and positions within the morphospace correspond to characteristic aspects of network topology (figure 1*a*). Specifically, in this paper we define a three-dimensional morphospace with axes given by *E*_diff_, *E*_rout_ and *C*_N_; therefore, networks are placed within the morphospace according to the previously defined measures.

In the context of this study, a morphospace exploration is the process of simulating brain-like networks and identifying their locations within the efficiency-complexity morphospace by measuring their corresponding values of *E*_diff_, *E*_rout_ and *C*_N_. Simulated brain-like networks are generated by incremental rewiring of a population of minimally perturbed empirical brain networks, which is implemented through a multi-objective evolutionary algorithm that approximates a Pareto-optimal set within the morphospace.

Given that the brain networks constructed from each dataset display differences in size and density, we define three distinct morphospaces, one for each dataset [46]. Within each morphospace, we constrained the set of possibly simulated networks to preserve the number of nodes and edges, degree sequence and network cost invariant with respect to the values measured from the corresponding empirical network. Finally, graph metrics are normalized in each morphospace with respect to the values of the corresponding empirical networks. Therefore, each empirical network is located within its morphospace in the coordinates (*E*_diff_, *E*_rout_, *C*_N_) = (1, 1, 1).

### (e) Morphospace analysis

In a theoretical morphospace, there is a distinction between possible and impossible forms (topologies), and a second distinction between functional and non-functional forms (topologies). The former distinction refers to topologies that are impossible because the combination of parameters (structural traits) is meaningless or infeasible and no network can satisfy such combination. The later distinction defines a subset of the formally possible topologies, which consists of the functionally feasible topologies. This means that within the space of possible topologies, there are networks that are not functionally feasible and therefore, such networks will not be found in the real world. For instance, all disconnected networks belong to the set of impossible brain network topologies. In addition, crucial properties of brain networks such as density, length and volume of neuronal connections, are subject to functional constraints that define the subset of biologically feasible networks within the set of possible networks in the brain network morphospace. In our work, we perform a morphospace exploration that implements functional constraints through a rewiring algorithm, whose objective is to preserve the total cost of the network connections. This strategy of morphospace exploration is based on evolving a population of networks by repeatedly carrying out two steps: network selection and network variation.

*Network selection* occurs according to Pareto optimality, a concept used in economics and engineering to describe a set of solutions that optimize multiple objectives simultaneously [47]. In general, a solution is said to be Pareto-optimal if an improvement of any single objective cannot be achieved without negatively affecting some other objective (figure 1*b*). In the context of a population or ensemble of networks that are being evaluated by multiple objective functions, a network *G* belongs to the Pareto-front set if and only if (1) *G* is not worse than any other network within the population, with respect to all objectives; (2) *G* is strictly better than any other network in the population, with respect to at least one objective [45] (figure 1*c*).

*Network variation* refers to small structural changes that are implemented with a rewiring algorithm. The algorithm is based on a random rewiring algorithm whose elementary moves are the so-called ‘edge-swaps’ [48]. An edge swap consists of the following steps:

(a) Randomly, select four distinct nodes, namely (*i*, *j*, *k*, *l*).
(b) If 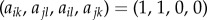 then swap the edges, so that the adjacency matrix entries become 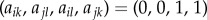.
(c) Otherwise, go back to (a).

This rewiring procedure ensures that the rewired network always remains connected and that the degree of each node is unchanged [49]. In this study, we require that each edge swap satisfies two additional conditions. The first condition is that the total wiring cost of the network must remain constant. The second condition is that the value of the fibre densities of all connections remains confined to the interval (0, *w*_max_), where *w*_max_ is the maximum fibre density found among all the connections of the empirical network. Therefore, when an edge swap is performed, the fibre densities *w_il_*, *w_jk_* corresponding to the added edges {*i*, *l*} and {*j*, *k*} are randomly chosen, provided that they satisfy the equation 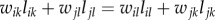 subject to 0 < *w_il_*, *w_jk_* < *w*_max_ (figure 1*d*). We refer to an edge swap that satisfies these conditions as a *rewiring step*.

#### (i) Fibre-length interpolation

Note that many times, when a pair of edges are swapped, the values of *l_il_* and *l_jk_* are not defined in the original connectivity matrices extracted from the neuroimaging data, simply because there is no actual connection between the pairs of nodes {*i*, *l*} and {*j*, *k*}. To assign fibre length values to edges created during the rewiring process, for each dataset a fully connected matrix *LI* was constructed combining existing and interpolated fibre length values between all pairs of nodes. This was done by making the assumption that two fibres whose starting and ending points are close in space should follow similar trajectories in the brain and thus have similar lengths. Under this assumption, an estimated fibre length based on similar existing fibres can be assigned to pairs of nodes that are not connected in the empirical network. We consider two fibres to be similar if we can define two neighbourhoods—one containing the starting nodes of the fibres, and another containing the ending points of the fibres—such that the radius of each neighbourhood is smaller than *δ*, where *δ* is defined as 20% of the Euclidean distance that separates the centres of both neighbourhoods. For every pair of unconnected nodes {*i*, *j*} in the empirical network, we defined such neighbourhoods and looked for fibres whose endpoints are in each of the neighbourhoods. If such fibres were found, we assigned their average length to the length of a fibre between nodes *i*, *j*. Finally, for all pairs of nodes for which we could not find similar fibres connecting their neighbourhoods, we used a polynomial interpolation of degree 2 to fit the fibre lengths as a function of Euclidean distance. For the right hemisphere sub-network of each dataset used in this study, the fraction of fibre lengths estimated by polynomial interpolation was 88%, 87% and 78% for LAU1, LAU2 and UTR respectively; the correlation between the interpolated fibre length matrix and the Euclidean distance matrix is 0.980, 0.982 and 0.992 for LAU1, LAU2 and UTR, respectively. Figure 1*e* shows the matrix of Euclidean distances between all pairs of nodes of the LAU1 network, together with the full fibre length matrix *LI* after the interpolation process.

#### (ii) Evolutionary process

All morphospace explorations start with an initial population of *M*_0_ = 500 networks, derived from the empirical brain network by performing three rewiring steps. During the evolutionary process, all networks preserve the number of nodes and edges, degree sequence and network cost of the corresponding empirical network. For every epoch of the evolution (defined by a single iteration of network selection, followed by network variation), the objective functions are evaluated on all of the population members. Selection according to Pareto optimality is applied to define the set of networks that pass unchanged to the next epoch. The set of networks that do not belong to the Pareto front are eliminated and substituted with a random sample of the Pareto-front members, which is subjected to minimum variation by carrying out one rewiring step on each network.

We avoid falling in to local maxima by introducing noise in the evolutionary process as follows. At a given epoch, if more than 90% of the population belongs to the Pareto-front set, then half of the population is randomly selected and subjected to one rewiring step. Then the simulation carries on.

In order to explore a greater extent of the sub-region of the morphospace surrounding the empirical brain network, we carried out eight independent runs of the evolutionary process. All runs start with the same initial population but implement distinct objective functions that aimed to drive the population of simulated brain networks towards the eight quadrants of the three-dimensional morphospace. The eight objective functions are defined as follows:

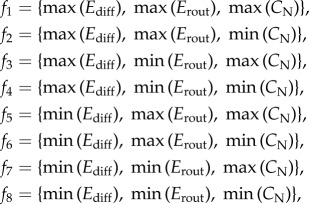

where max() and min() stand for the maximization and minimization function. The maximum number of iterations (epochs) for each objective function was set to 2000; however, all eight runs of the evolutionary process (one per objective function) required different CPU times to compute, and CPU times varied depending on the objective functions and the datasets. Therefore, the stopping condition for each evolutionary process was either 2000 iterations completed or 7 days of computation.

It is worth mentioning that in this work, the terms *evolution* and *selection pressure* are used within the context of a computational algorithm, and their usage does not necessarily reflect a direct mapping onto processes studied in the field of evolutionary biology.

## 3. Results

### (a) Randomization and latticization of the brain networks

We explored the behaviour of the efficiency and complexity measures when the empirical networks are rewired towards two canonical models, namely a spatial lattice-like network and a random network. Both processes involving the randomization and latticization of the empirical networks use the edge swapping algorithm (see §2*e*) iteratively to rewire the networks; this guarantees that the *latticized* and *randomized* networks preserve the number of nodes and edges, degree sequence and wiring cost. The latticization process takes into account the spatial positions (Euclidean distances) of the network nodes in order to create a lattice-like network where nodes tend to be connected to their spatially nearest neighbours.

**Figure 2.**
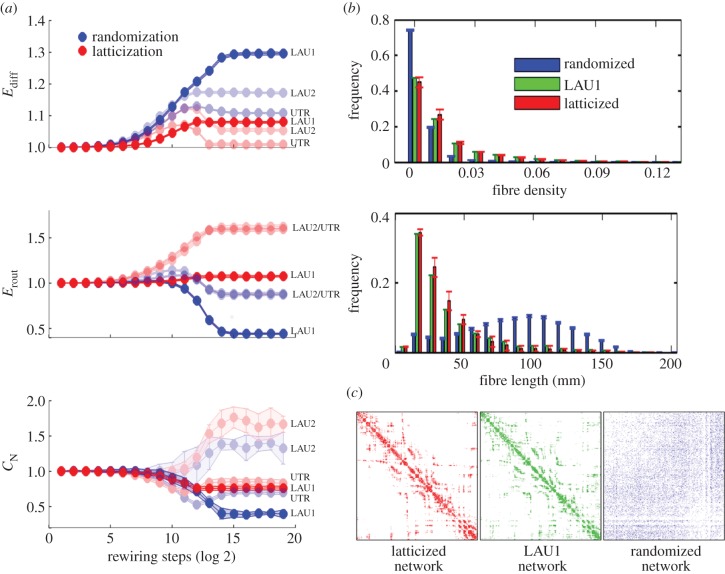
(*a*) Latticization and randomization of empirical brain networks. (*b*) Fibre length and fibre density distributions of randomized networks (blue), LAU1 network (green) and latticized networks (red). Randomized and latticized network distributions are averages over 40 repetitions of the randomization and latticization process applied to the LAU1 empirical network. (*c*) Adjacency matrices of latticized LAU1 network (red), LAU1 network (green) and randomized LAU1 network (blue).

Figure 2*a* shows *E*_diff_, *E*_rout_ and *C*_N_ as a function of the number of rewiring steps carried out on all three empirical networks during their randomization (blue dots) and latticization (red dots), respectively. All values are averages over 40 repetitions of the randomization and the latticization processes.

In all three datasets, the three measures reach a stable regime after 2^15^ rewiring steps, suggesting that additional rewiring steps do not further change the topology of the networks. Qualitatively, the efficiency measures behave similarly across the three datasets during the randomization process: *E*_diff_ increases while *E*_rout_ decreases as a function of the number of rewiring steps towards randomization. *C*_N_ decreases in the LAU1 and UTR datasets, in agreement with earlier studies showing that randomly rewiring cortical networks tends to decrease their complexity [14]. However, we found inconsistent behaviour of *C*_N_ for the LAU2 network, as well as larger variance of *C*_N_ across repetitions of the randomization and latticization. In order to test whether the observed variability of *C*_N_ is intrinsic to the LAU2 network or if it is produced by the approximation method used to calculate *C*_N_ (see §2*c*(iii)), we computed *C*_N_ values of randomized and latticized networks with the *sampling method* (see §2*c*(iii)); the correlation between approximated and sampled *C*_N_ values is 0.989 (*p* < 0.01) for the randomized networks and 0.995 (*p* < 0.01) for the latticized networks, thus suggesting that variability in *C*_N_ is due to differences in network topology across the datasets.

An interesting finding is that randomization decreases *E*_rout_. By contrast, high *E*_rout_ is a typical characteristic of random networks [9] when these networks are not constrained to preserve network cost. The study of the fibre length and fibre density distributions of the empirical, randomized and latticized networks (figure 2*b*) reveals topological changes that have an effect on communication efficiency. While randomization tends to increase the amount of long-range connections in the networks, it also has the effect of decreasing the fibre density of the majority of the connections; overall, such thinning of connections diminishes *E*_rout_. Regarding the observed increase of *E*_rout_ during the latticization, we note that latticized networks tend to have higher fibre density on short-distance connections, which promotes shorter path length and thus favours *E*_rout_.

### (b) The effects of minimal perturbations on the structure of brain networks

**Figure 3.**
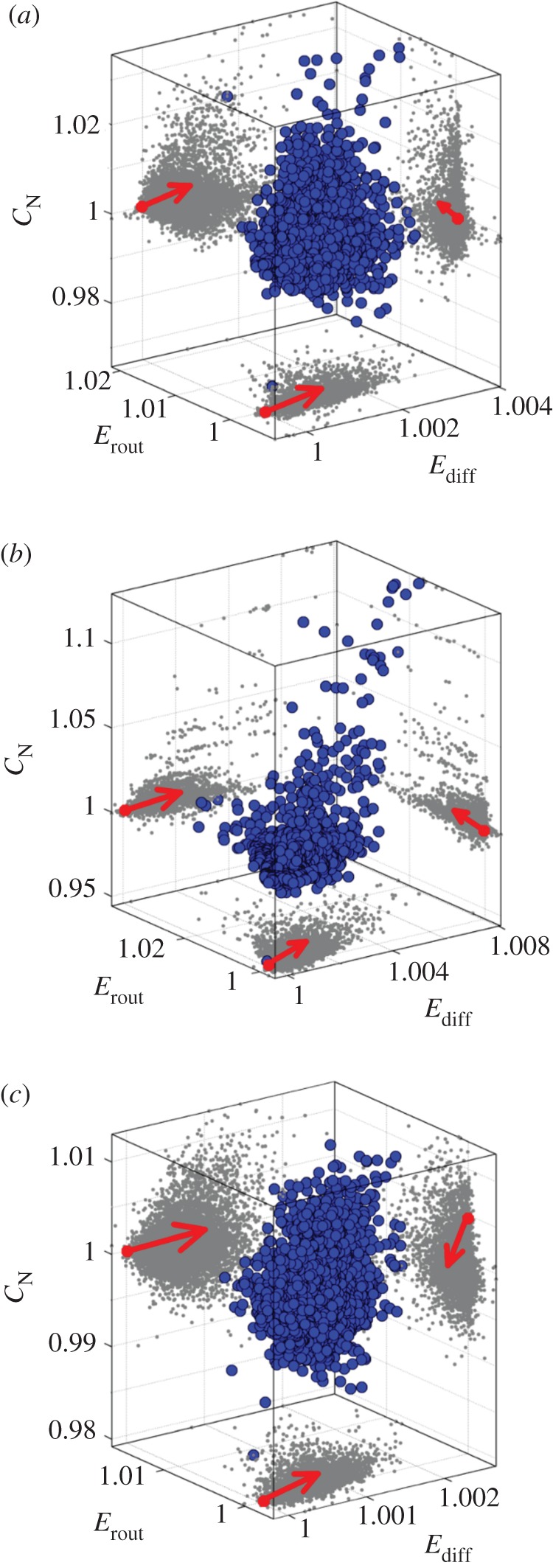
Population of proximal network elements of (*a*) LAU1, (*b*) LAU2 and (*c*) UTR networks, respectively. Every blue dot shows the location of a network that was created by applying three rewiring steps to the respective empirical network. Grey dots show the two-dimensional projections onto the distinct planes of the morphospace. Red dots at the origin of the arrows indicate the projections in each two-dimensional plane of the three-dimensional coordinates (1, 1, 1), which correspond to the location of the empirical networks within the respective morphospace. Arrows point towards the preferred direction in which proximal networks are located in each plane.

The second aim of our work is to characterize the effects of small perturbations on the structure of brain networks. To do so, we generated three populations of 10 000 minimally rewired network variants, each created by carrying out three rewiring steps on each of the three empirical networks, LAU1, LAU2 and UTR. We used three rewiring steps because it is the minimum number of rewiring steps that allows us to distinguish numerical differences in all three dimensions of the morphospace. In this way, we explore the proximal morphospace, that is, the space that contains the closest neighbouring network elements of each empirical network. Interestingly, in all three datasets we found that 99% of the neighbouring networks are in a region of the morphospace defined by *E*_diff_ > 1, i.e. most networks have higher values of *E*_diff_, compared with their corresponding empirical networks. This suggests that the three empirical networks are located very close to a (local) *E*_diff_ minimum and that the region of morphospace defined by *E*_diff_ ≤ 1 is difficult to access, given the topological constraints imposed by the rewiring algorithm (see §2*d*,*e*). The proportion of networks contained in the region *E*_rout_ > 1 and *C*_N_ > 1 varied across datasets: 35.26%, 85.14% and 75.22% of the populations extracted from the LAU1, LAU2 and UTR datasets, respectively, were in the region *E*_rout_ > 1; 28.21%, 16.07% and 27.25% of the populations (LAU1, LAU2 and UTR, respectively) were in the region *C*_N_ > 1; finally, 9.66%, 14.59% and 21.92% of the populations were in the region {*E*_diff_ > 1, *E*_rout_ > 1, *C*_N_ > 1} of the respective morphospace (LAU1, LAU2 and UTR). Figure 3 shows the distributions of proximal networks embedded in the three morphospaces corresponding to each dataset. The shape of the region occupied by these networks shows that the accessibility of the sub-region of morphospace surrounding the coordinates (*E*_diff_, *E*_rout_, *C*_N_) = (1, 1, 1) is not uniform and that there are ‘preferred’ directions along each axis in which networks are located.

### (c) Exploring the efficiency-complexity morphospace of brain networks

We implemented an evolutionary algorithm to explore a sub-region of the efficiency-complexity morphospace in search for alternative biologically feasible brain-like networks (see §2*e*(ii)). In order to characterize the structural traits of the networks simulated through distinct selection pressures, we used eight objective functions to drive a population of networks towards the eight quadrants of the three-dimensional morphospace. For each dataset, we carried out 10 repetitions of the exploratory process. Each repetition of the process includes: (i) creating an initial population of 500 networks by minimally rewiring the empirical network and (ii) applying the evolutionary process eight times independently, once for each objective function (see §2*e*(ii)). Therefore, the completion of an exploratory process yields eight distinct populations of 500 networks that have been subject to different selection pressures. We will refer to each one of these populations as a front: front 1 is the population evolved by optimizing the objective function *f*_1_; front 2 is the population evolved by optimizing *f*_2_, and so on. The stopping condition for the evolutionary process was either 2000 iterations completed or 7 days of computation. During the 10 repetitions of the morphospace exploration, for the datasets LAU1 and LAU2, the evolutionary process of all eight fronts was stopped after 2000 iterations. For the UTR dataset, the evolutionary process of fronts 3, 5, 6, 7 and 8 completed 2000 iterations, while the evolution of fronts 1, 2 and 4 was stopped after 7 days of computation, during which the processes had simulated on average 1460, 1315 and 1670 iterations, respectively. Note that all networks belonging to any front are embedded in a sub-region of the morphospace that is still fairly close to the empirical brain network; structural changes in the simulated networks account for, on average, 20% and 8% of the total number of edges present in the networks in fronts 1 through 4 and fronts 5 through 8, respectively.

**Figure 4.**
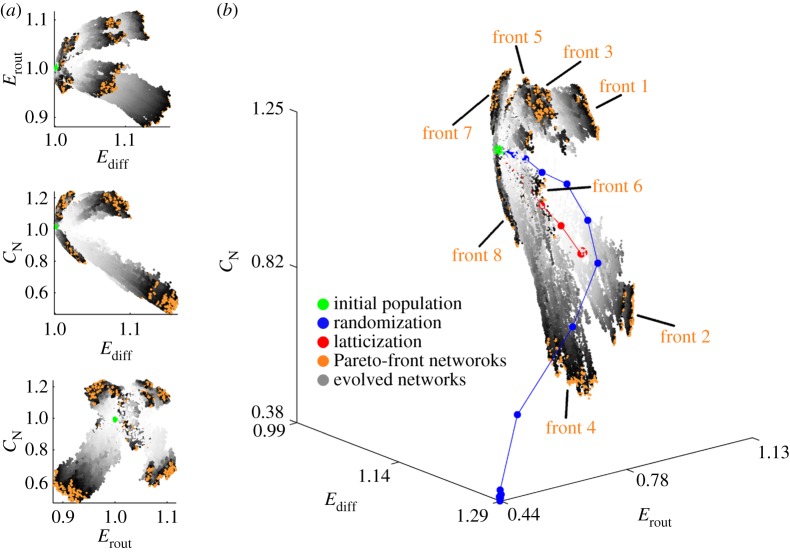
Evolved brain networks located within the LAU1 efficiency-complexity morphospace. (*a*) Two-dimensional projections of the three-dimensional morphospace. The coordinates (*E*_diff_, *E*_rout_, *C*_N_) = (1, 1, 1) are the coordinates corresponding to the LAU1 network, and therefore the initial population is located very close to those coordinates (cf. figure 3). All points indicate the regions of morphospace explored by eight independent runs of the optimization algorithm, all starting with the same initial population but driven by eight distinct objective functions (see §2*e*(ii)). The grey-scale assigned to each network indicates the epoch in which it was created, with light grey corresponding to early epochs and darker grey to later epochs. Orange points correspond to the Pareto-front networks of the last epoch of each front. (*b*) Three-dimensional efficiency-communication morphospace. Blue and red points show the average trajectory of a randomized and latticized brain network, respectively, which are not subjected to the selective pressures imposed when exploring the different fronts. The grey-scale assigned to each network indicates the epoch in which it was created, with light grey corresponding to early epochs and darker grey to later epochs. Orange points correspond to the Pareto-front networks of the last epoch of each front.

Figure 4 shows the regions of the morphospace explored during the evolution of the eight fronts, starting with a population of networks derived from the LAU1 network (see the electronic supplementary material, figures S1 and S2, for LAU2 and UTR datasets, respectively). Although the shape and extent of the regions explored by each evolving front vary across datasets, we find three important aspects that are consistent, regardless of the dataset used to derive the initial population. First, the evolutionary algorithm is unable to find solutions within the region defined by {*E*_diff_ < 1}. Second, none of the fronts follows the trajectory of a randomization or latticization process. This demonstrates that the evolution of the network populations towards different regions of the morphospace is driven by distinct selection pressures, and not by the random nature of the rewiring algorithm. Third, the evolutionary process is able to generate brain-like networks within the region {*E*_diff_ > 1, *E*_rout_ > 1, *C*_N_ > 1}; that is, all three topological aspects of brain networks can simultaneously increase, while preserving wiring cost.

### (d) Characterization of Pareto-optimal brain networks

**Figure 5.**
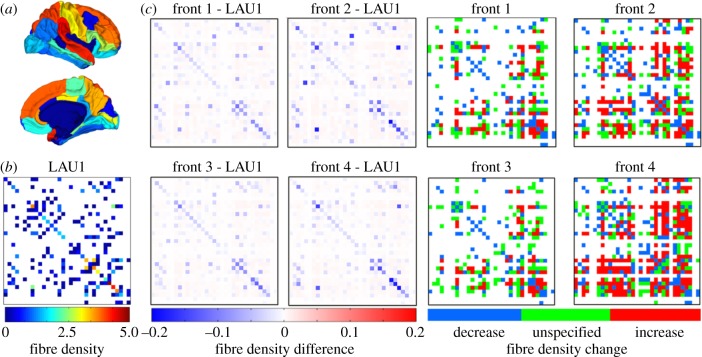
Examples of low-resolution brain networks. (*a*) Low-resolution partition of the right hemisphere of the human cortex, composed of 33 anatomical areas. (*b*) Down-sampled connection weights (fibre densities) of LAU1 network. (*c*) Difference between the average networks of fronts 1 through 4, and the LAU1 network. Blue elements indicate negative sign; red elements indicate positive sign. (*d*) Consistent changes of connection strengths in evolved network populations: colours indicate whether fibre densities increased (red), decreased (blue) or changed in an unspecific direction (green) across 90% or more of the evolved networks belonging to one front.

To allow comparisons across datasets, brain networks were down-sampled into a commonly used low-resolution partition of the human cortex, composed of 66 anatomical areas [50], with 33 areas representing the right cortical hemisphere of the brain (see figure 5*a*). For each dataset, networks evolved towards eight fronts (10 repetitions per front) and the final populations of 500 evolved networks were down-sampled to the low-resolution partition and then aggregated according to front membership. Thus, we obtained eight populations (one for each front) of low-resolution brain networks, each population containing 5000 evolved brain networks. As the four fronts driving networks towards {*E*_diff_ < 1} failed to advance, their evolved networks were not investigated further. For the remaining four fronts, we identified anatomical pathways whose fibre density and/or cost has significantly changed during the evolutionary process to favour particular topological traits. For each front, all final populations of evolved networks were aggregated into a single average network, representative of the corresponding front. The differences between the average networks of each front and the corresponding empirical network are shown in figure 5*c*, together with the corresponding plots recording the consistency with which connections increased or decreased in strength (figure 5*d*). Each of the fronts is associated with a characteristic pattern of changes in connection weights, and visual inspection suggests greater similarity in the patterns for fronts 1 and 3, and for patterns for fronts 2 and 4, respectively. Analysis of the pairwise cosine angles between average networks of each front confirms this observation, with fronts 1 and 3 (both maximizing *C*_N_) and fronts 2 and 4 (both minimizing *C*_N_) exhibiting the greatest similarity across all three datasets.

**Figure 6.**
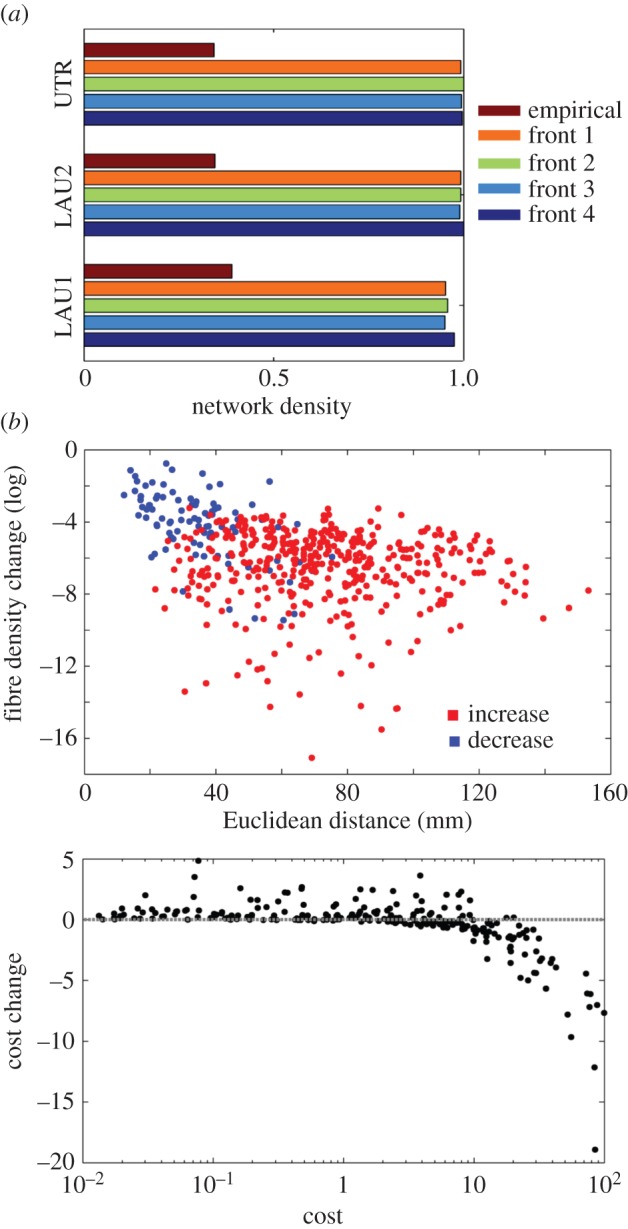
Consistent changes in connection patterns observed in fronts 1 through 4 across all three datasets. (*a*) Density of empirical and evolved networks. (*b*) New connections (red points) in evolved networks tend to extend over long spatial distances; fibre densities that are weakened during evolution (blue points) tend to involve pairs of nodes that are spatially close or belong to the same anatomical region. (*c*) High-cost connections are principal targets for rewiring during the evolutionary process.

Other aspects of changes in connection patterns were consistently observed across all three datasets. First, the density of evolved networks in all four fronts increases significantly (figure 6*a*), indicating that areas originally unconnected have a strong tendency to become weakly connected. These new projections appear as a result of rewiring of edges away from denser pathways, thus sculpting their overall pattern into a new topology and steering the population towards one of the four Pareto fronts. Second, most of these newly formed projections extend over long spatial distances, while most of the projections that become weakened involve nodes that are spatially close, including node pairs that belong to the same anatomical region (figure 6*b*). Finally, a cost analysis suggests that high-cost connections, i.e. connections that contribute strongly to the overall cost of the network (which is conserved in our simulations) are principal targets for rewiring (figure 6*c*). Their rewiring results in a dispersal of their contribution to network cost to a larger set of connections spanning a greater number of anatomical regions.

## 4. Discussion

In this work, we applied a multi-objective evolutionary approach to rewire brain networks and place them within an efficiency-complexity morphospace. Using various Pareto-optimal selection criteria, we were able to explore how brain networks evolve when subject to distinct selection pressures. Furthermore, the approach allowed us to investigate relationships and trade-offs between distinct topological traits associated with the principal axes of the morphospace, *E*_diff_, *E*_rout_ and *C*_N_. Our results demonstrate that the empirical networks we used as seed points for evolution are surrounded by a large space of variant network topologies, even when holding wiring cost constant, including networks that combine a higher capacity to support efficient communication with higher neural complexity.

Our work attempts to make several methodological contributions. First, building on fundamental work in evolutionary theory [44,51,52], we extend the morphospace analysis framework into the realm of human brain networks. In the past, morphospace analysis has been applied independently in the field of complex networks [11,53,54], and in the field of neuroscience [55]; here, we combine methodology from the three fields, evolutionary biology, neuroscience and complex networks to study the topological features available for biologically feasible brain-like networks. Our work proposes that differences among variants of human brain networks can be investigated by placing these variants into a space formed by several principal axes representing fundamental measures of network organization. Within this space, gradual rewiring of network nodes and edges, for example by applying multi-objective optimization, ‘moves’ networks towards new topological patterns.

Second, as previously introduced in [11], here we explored two separate measures of network efficiency, one based on communication along shortest paths (routing-based communication) [9] and the other based on diffusion processes. In addition, we considered a dynamic measure of neural complexity that expressed the coexistence of segregation and integration in the network [12].

Third, as we were interested in the trade-off of these efficiency and complexity measures within the cost constraints imposed by human brain size and geometry, we employed a rewiring rule that conserved not only node degree [48,49] but also overall network cost, by adjusting the fibre density of rewired connections according to their wiring length (figure 1*d*).

Our first interesting finding was that full randomization resulted in networks that were less efficient in routing communication. Notably, this result diverged from the sharp increase in routing efficiency observed when non-cost-conserving randomization models are applied. When conserving cost, randomizing brain connectivity is necessarily accompanied by a thinning out of the fibre densities (shown in the distribution of the fibre density of randomized networks, figure 2), because most randomized connections span greater distances; such low-density connections do not contribute towards efficient routing communication. Full latticization of brain networks does not produce perfect lattices because of the constraints imposed by the rewiring rule; in fact, a comparison of the fibre length and density distributions between the latticized networks and the respective empirical networks reveals great similarity between these networks. Latticized networks differ slightly from the empirical networks in that they tend to have higher fibre density on short-distance connections, which promotes shorter path length and thus favours *E*_rout_.

Our next results were derived from studying the effects that minimal structural perturbations have over the measures of communication efficiency and dynamical complexity. The accessibility of the proximal morphospace (the region immediately surrounding the empirical networks) was found to be non-uniform, e.g. many more network variants exhibited greater *E*_rout_ and *E*_diff_. This result held for all three datasets.

A local exploration of the efficiency-complexity morphospace confirmed that, given the network invariants of cost, density and degree sequence, the morphospace region *E*_diff_ < 1 is restricted, and thus it is all but impossible to further decrease diffusion efficiency in all three datasets. Four separate fronts failed to advance in the direction of *E*_diff_ < 1, suggesting that the topology of empirical brain networks tends to minimize *E*_diff_. Most important, we note that three different concepts play into the discussion of this result. First, what is the relative magnitude of a network's *E*_diff_ (with respect to a null model) and to what extent can *E*_diff_ be decreased (increased) through a rewiring process that preserves certain network features? Second, to what extent can the dynamics occurring on top of a network structure be explained or predicted by a diffusion-based model, regardless of whether diffusion-like dynamics are an efficient communication scheme for the system? Third, to what extent have diffusion-based dynamics been a critical evolutionary pressure shaping the structure of brain networks?

The relative value of *E*_diff_ captures to what degree the structure of the network facilitates the integration of information when information spreads through diffusion-based dynamics. Therefore, in this paper, we can answer the first question by providing evidence that brain networks are close to a minimum of *E*_diff_ (of course, within the space of networks with a fixed number of nodes and connections, a predetermined degree sequence and an invariant global connection cost). Furthermore, we can conclude that the topology of structural brain networks does not facilitate an efficient communication between all pairs of nodes, provided that information within the network spreads solely as a diffusion process. However, the relative value of a network's *E*_diff_ does not provide any information about the underlying process through which information actually spreads within the network. Other studies have used different approaches to address this question; for example, Betzel *et al.* [56] and Goñi *et al.* [57] have provided evidence that brain dynamics can be modelled and/or explained by diffusion-like processes. It is important to bear in mind that in these studies diffusion-like processes were used as models for the spreading of perturbations (as generative models for resting-brain functional connectivity), and it is well known from studies in other systems that modular topologies tend to limit the spread of perturbations across module boundaries. Finally, in this paper we can only speculate about the third question, regarding the role of diffusion-based dynamics as an evolutionary pressure. Nonetheless, in our opinion, it seems very unlikely that *E*_diff_ is not a critical evolutionary factor shaping brain networks given that the value of *E*_diff_ that brain networks exhibit is not arbitrary, but is actually at a minimum. One possible interpretation is that minimizing *E*_diff_ has been due to critical evolutionary pressure shaping brain network topology in order to limit passive diffusion (e.g. of noisy perturbations) on global scales while promoting efficient diffusive communication on local scales, such as within network communities [56].

Conversely to the severe constraints found on the *E*_diff_ axis, we did not find such strict constraints on the other two axes of the efficiency-complexity morphospace. Greater *E*_rout_ as well as greater *C*_N_, singly or in combination, could be achieved through rewiring of specific pathways in all three datasets. Fronts advancing towards greater or lesser *C*_N_ exhibited greater consistency in rewiring of specific pathways, suggesting that neural complexity depends more strongly on specific network topologies. Instead, there appear to be more structural configurations available for networks belonging to the fronts advancing towards higher or lower *E*_rout_.

In addition to these trends that were specific to the multi-objective function employed, we also observed some aspects of the rewiring process that were shared among all fronts. These aspects included a strong tendency to create new (albeit weak) pathways linking previously unconnected anatomical regions by redistributing connections away from node pairs that were spatially close and/or linked by high-cost connections. This study does not allow us to determine whether these general tendencies mainly reflect constraints imposed by the cost-conserving rewiring rule or if they point to greater accessibility of parts of the morphospace by structural network variants that are more diffusely or densely connected. We acknowledge that there are several methods to rewire weighted networks and that the constraints imposed by any rewiring method will have certain effects on the topologies of the rewired networks. For instance, a rewiring algorithm that is constrained to preserve the distribution of connection weights will necessarily have the effect of increasing the total connection cost. This is because the rewiring process tends to create long-range connections and the cost measure used in this study is proportional to the connection lengths. Alternatively, to preserve crucial aspects of brain networks throughout the rewiring process, one could enforce preserving both the distribution of connection weights and the distribution of connection lengths. However, such a set of restrictions would have the effect of drastically reducing the space of solutions, imposing severe limitations on the exploration of the morphospace. Hence, for this study, we have opted for an approach that is not as restrictive as the latter but is still conservative, that is to preserve the total cost of the connections of the networks. This approach provides sufficient degrees of freedom to explore the morphospace, while imposing a strong functional constraint that allows us to study biologically feasible brain networks. Furthermore, the rewiring algorithm we present in this study provides an alternative null model to perform statistical tests of graph measures of brain networks. The use of random networks as null models is very common [46]; however, as we have pointed out previously, there are several ways to randomize a network, and hypothesis testing will yield different results depending on the selected null model. Here, we suggest that the appropriate null model is one that preserves most of a brain network's basic features that make it biologically feasible, such as grey/white matter volume, connection density and degree sequence, among others.

Several aspects of the present work require future extensions. First, networks derived from the three datasets employed here shared numerous topological features but were also somewhat variable due to differences in subject cohort, data acquisition and tractography (see §2*b*). These differences did not allow firm inferences about which changes in specific anatomical pathways were associated with specific Pareto fronts (i.e. due to specific selection pressures). This inference awaits the arrival of more uniformly acquired, normative datasets, for example those collected as part of the Human Connectome Project [58]. Second, while the current work examined the trade-off between different measures of network communication efficiency and complexity, the trade-off of these measures with network cost (held constant in this study) remained unexplored. This could be explored, for instance, by relaxing the invariant cost assumption used here and introducing network cost as a term in the objective function instead (either conforming or being part of one of the morphospace axes). Third, the measures forming the principal axes of the morphospace were chosen on the basis of previous work [11,12,14] which suggested that they are relevant for various aspects of brain function and dynamics; however, alternative formulations of network morphospace that target other features of network structure and topology may be explored in future work. Furthermore, as opposed to using explicit network measures, one could favour orthogonality or statistical independence (e.g. independent component analysis) when defining the morphospace axes and the topological invariants in the evolutionary algorithm. Finally, further extensions of this work may also include an analysis of local or within-community communication efficiency, because global measures do not capture how communication efficiency is distributed among network communities.

These and other extensions of the current work may become useful for characterizing regions of network morphospace that are occupied by existing topological variants of the human brain. As is the case for biological forms [44], we expect that the majority of the morphospace is empty, i.e. most possible network configurations are either physically or economically infeasible or have been selected against in evolution. Among those variants that do occur, we expect that embedding of individual human brains in network morphospace will highlight important patterns in individual differences of network organization, including those associated with disease-related network disturbances.

## Supplementary Material

Figure S1

## Supplementary Material

Figure S2

## Supplementary Material

Data set LAU1

## Supplementary Material

Data set LAU2

## Supplementary Material

Data set UTR

## References

[1] Sporns O 2011 Networks of the brain. Cambridge, MA: MIT Press.

[2] Park HJ Friston K 2013 Structural and functional brain networks: from connections to cognition. Science 342, 1238411 (10.1126/science.1238411) 24179229

[3] Hagmann P Cammoun L Gigandet X Meuli R Honey C Wedeen V Sporns O 2008 Mapping the structural core of human cerebral cortex. PLoS Biol. 6, e159 (10.1371/journal.pbio.0060159) 18597554 PMC2443193

[4] Gong G He Y Concha L Lebel C Gross D Evans AC Beaulieu C 2009 Mapping anatomical connectivity patterns of human cerebral cortex using *in vivo* diffusion tensor imaging tractography. Cereb. Cortex 19, 524–536. (10.1093/cercor/bhn102) 18567609 PMC2722790

[5] van den Heuvel MP Sporns O 2011 Rich club organization of the human connectome. J. Neurosci. 31, 15775–15786. (10.1523/JNEUROSCI.3539-11.2011) 22049421 PMC6623027

[6] Mitchison G 1991 Neuronal branching patterns and the economy of cortical wiring. Proc. R. Soc. Lond. B 245, 151–158. (10.1098/rspb.1991.0102) 1682939

[7] Cherniak C 1994 Component placement optimization in the brain. J. Neurosci. 14, 2418–2427. 8158278 10.1523/JNEUROSCI.14-04-02418.1994 PMC6577144

[8] Ahn Y Jeong H Kim B 2006 Wiring cost in the organization of a biological neuronal network. Phys. A 367, 551 (10.1016/j.physa.2005.12.013).

[9] Latora V Marchiori M 2001 Efficient behavior of small-world networks. Phys. Rev. Lett. 87, 198701 (10.1103/PhysRevLett.87.198701) 11690461

[10] Laughlin SB Sejnowski TJ 2003 Communication in neuronal networks. Science 301, 1870–1874. (10.1126/science.1089662) 14512617 PMC2930149

[11] Goñi J Avena-Koenigsberger A de Mendizabal NV van den Heuvel M Betzel R Sporns O 2013 Exploring the morphospace of communication efficiency in complex networks. PLoS ONE 8, e58070 (10.1371/journal.pone.0058070) 23505455 PMC3591454

[12] Tononi J Sporns O Edelman G 1994 A measure for brain complexity: relating functional segregation and integration in the nervous system. Proc. Natl Acad. Sci. USA 91, 5033–5037. (10.1073/pnas.91.11.5033) 8197179 PMC43925

[13] Friston KJ 2002 Beyond phrenology: what can neuroimaging tell us about distributed circuitry? Annu. Rev. Neurosci. 25, 221–250. (10.1146/annurev.neuro.25.112701.142846) 12052909

[14] Sporns O Tononi G Edelman GM 2000 Theoretical neuroanatomy: relating anatomical and functional connectivity in graphs and cortical connection matrices. Cereb. Cortex 10, 127–141. (10.1093/cercor/10.2.127) 10667981

[15] Laughlin SB de Ruyter van Steveninck RR Anderson JC 1998 The metabolic cost of neural information. Nat. Neurosci. 1, 36–41. (10.1038/236) 10195106

[16] Barthelemy M 2011 Spatial networks. Phys. Rep. 499, 1–101. (10.1016/j.physrep.2010.11.002)

[17] Henderson JA Robinson PA 2011 Geometric effects on complex network structure in the cortex. Phys. Rev. Lett. 107, 018102 (10.1103/PhysRevLett.107.018102) 21797575

[18] Chen B Hall D Chklovskii D 2006 Wiring optimization can relate neuronal structure and function. Proc. Natl Acad. Sci. USA 103, 4723–4728. (10.1073/pnas.0506806103) 16537428 PMC1550972

[19] Young MP 1992 Objective analysis of the topological organization of the primate cortical visual system. Nature 358, 152–155. (10.1038/358152a0) 1614547

[20] Klyachko VA Stevens CF 2003 Connectivity optimization and the positioning of cortical areas. Proc. Natl Acad. Sci. USA 100, 7937–7941. (10.1073/pnas.0932745100) 12796510 PMC164691

[21] Markov NT 2013 The role of long-range connections on the specificity of the macaque interareal cortical network. Proc. Natl Acad. Sci. USA 110, 5187–5192. (10.1073/pnas.1218972110) 23479610 PMC3612613

[22] Kaiser M Hilgetag C 2006 Nonoptimal component placement, but short processing paths, due to long-distance projections in neural systems. PLoS Comput. Biol. 2, e95 (10.1371/journal.pcbi.0020095) 16848638 PMC1513269

[23] Sporns O Honey CJ Kotter R 2007 Identification and classification of hubs in brain networks. PLoS ONE 2, e1049 (10.1371/journal.pone.0001049) 17940613 PMC2013941

[24] Newman MEJ 2006 Modularity and community structure in networks. Proc. Natl Acad. Sci. USA 103, 8577–8582. (10.1073/pnas.0601602103) 16723398 PMC1482622

[25] Meunier D Lambiotte R Bullmore ET 2010 Modular and hierarchically modular organization of brain networks. Front. Neurosci. 4, 200 (10.3389/fnins.2010.00200) 21151783 PMC3000003

[26] Watts DJ Strogatz SH 1998 Collective dynamics of ‘small-world’ networks. Nature 393, 440–442. (10.1038/30918) 9623998

[27] Bassett DS Bullmore E 2006 Small-world brain networks. Neuroscientist 12, 512–523. (10.1177/1073858406293182) 17079517

[28] Achard S Bullmore E 2007 Efficiency and cost of economical brain functional networks. PLoS Comput. Biol. 3, e17 (10.1371/journal.pcbi.0030017) 17274684 PMC1794324

[29] Deco G Jirsa VK McIntosh AR 2013 Resting brains never rest: computational insights into potential cognitive architectures. Trends Neurosci. 36, 268–274. (10.1016/j.tins.2013.03.001) 23561718

[30] Sporns O 2013 Network attributes for segregation and integration in the human brain. Curr. Opin. Neurobiol. 23, 162–171. (10.1016/j.conb.2012.11.015) 23294553

[31] Bullmore E Sporns O 2012 The economy of brain network organization. Nat. Rev. Neurosci. 13, 336–349. 22498897 10.1038/nrn3214

[32] Newman M 2003 The structure and function of complex networks. SIAM Rev. 45, 167–256. (10.1137/S003614450342480)

[33] Rubinov M Sporns O 2010 Complex network measures of brain connectivity: uses and interpretations. Neuroimage 52, 1059–1069. (10.1016/j.neuroimage.2009.10.003) 19819337

[34] Bullmore ET Bassett DS 2011 Brain graphs: graphical models of the human brain connectome. Annu. Rev. Clin. Psychol. 7, 113–140. (10.1146/annurev-clinpsy-040510-143934) 21128784

[35] Daducci A Gerhard S Griffa A Lemkaddem A Cammoun L Gigandet X Meuli R Hagmann P Thiran J-P 2012 The connectome mapper: an open-source processing pipeline to map connectomes with MRI. PLoS ONE 7, e48121 (10.1371/journal.pone.0048121) 23272041 PMC3525592

[36] Cammoun L Gigandet X Meskaldji D Thiran J Sporns O Do K Maeder P Meuli R Hagmann P 2012 Mapping the human connectome at multiple scales with diffusion spectrum MRIJ. Neurosci. Methods 203, 386–397. (10.1016/j.jneumeth.2011.09.031) 22001222

[37] Wedeen VJ 2008 Diffusion spectrum magnetic resonance imaging (DSI) tractography of crossing fibers. Neuroimage 41, 1267–1277. (10.1016/j.neuroimage.2008.03.036) 18495497

[38] van den Heuvel MP 2013 Abnormal rich club organization and functional brain dynamics in schizophrenia. JAMA Psychiatry 70, 783–792. (10.1001/jamapsychiatry.2013.1328) 23739835

[39] Jbabdi S Johansen-Berg H 2011 Tractography: where do we go from here? Brain Connect. 1, 169–183. (10.1089/brain.2011.0033) 22433046 PMC3677805

[40] Grinstead CCM Snell JL 1997 Introduction to probability, 2nd edn Providence, RI: American Mathematical Society.

[41] Barnett L Buckley CL Bullock S 2009 Neural complexity and structural connectivity. Phys. Rev. E Stat. Nonlin. Soft Matter Phys. 79, 051914 (10.1103/PhysRevE.79.051914) 19518487

[42] Galan RF 2008 On how network architecture determines the dominant patterns of spontaneous neural activity. PLoS ONE 4, e2148 (10.1371/journal.pone.0002148) PMC2374893 18478091

[43] Sporns O Tononi G 2002 Classes of network connectivity and dynamics. Complexity 7, 28–38. (10.1002/cplx.10015)

[44] McGhee GR 1999 Theoretical morphology: the concept and its applications. New York, NY: Columbia University Press.

[45] Petrie C Webster T Cutkosky M 1995 Using Pareto optimality to coordinate distributed agents. AIEDAM 9, 269–281. (10.1017/S0890060400002821)

[46] Van Wijk BCM Stam CJ Daffertshofer A 2010 Comparing brain networks of different size and connectivity density using graph theory. PLoS ONE 5, e13701 (10.1371/journal.pone.0013701) 21060892 PMC2965659

[47] Goldberg DE 1989 Genetic algorithms in search, optimization and machine learning. Boston, MA: Addison-Wesley Longman.

[48] Coolen ACC De Martino A Annibale A 2009 Constrained Markovian dynamics of random graphs. J. Stat. Phys. 136, 1035–1067. (10.1007/s10955-009-9821-2)

[49] Maslov S Sneppen K 2002 Specificity and stability in topology of protein networks. Science 296, 910–913. (10.1126/science.1065103) 11988575

[50] Fischl B 2004 Automatically parcellating the human cerebral cortex. Cereb. Cortex 14, 11–22. (10.1093/cercor/bhg087) 14654453

[51] Shoval O Sheftel H Shinar G Hart Y Ramote O Mayo A Dekel E Kavanagh K Alon U 2012 Evolutionary trade-offs, pareto optimality, and the geometry of phenotype space. Science 336, 1157–1160. (10.1126/science.1217405) 22539553

[52] Esteva-Altava E Rasskin-Gutman D 2014 Theoretical morphology of tetrapod skull networks. CR Palevol. 13, 41–50. (10.1016/j.crpv.2013.08.003)

[53] Ferrer i Cancho R Solé R 2003 Optimization in complex networks. In Statistical mechanics of complex networks (eds Pastor-Satorras R Rubi M Diaz-Guilera A), pp. 114–126. Berlin, Germany: Springer.

[54] Corominas-Murtra B Goñi J Solé R Rodríguez-Caso C 2013 On the origins of hierarchy in complex networks. Proc. Natl Acad. Sci. USA 110, 13 316–13 321. (10.1073/pnas.1300832110) PMC3746874 23898177

[55] Costa Lda F Zawadazki K Miazaki M Viana MP Tarasakin SN 2010 Unveiling the neuromorphological space. Front. Comput. Neurosci. 4, 150. 21160547 10.3389/fncom.2010.00150 PMC3001740

[56] Betzel RF Griffa A Avena-Koenigsberger A Goñi J Thiran J-P Hagmann P Sporns O 2014 Multi-scale community organization of the human structural connectome and its relationship with resting-state functional connectivity. Network Sci. 1, 353–373. (10.1017/nws.2013.19)

[57] Goñi J 2013 Resting-brain functional connectivity predicted by analytic measures of network communication. Proc. Natl Acad. Sci. USA 111 833–838. (10.1073/pnas.1315529111) 24379387 PMC3896172

[58] Van Essen D Smith SM Barch DM Behrens TEJ Yacoub E Ugurbil K 2013 The WU-Minn human connectome project: an overview. Neuroimage 80, 62–79. (10.1016/j.neuroimage.2013.05.041) 23684880 PMC3724347

