# Supplementary figures and images for: Using Pareto optimality to explore the topology and dynamics of the human connectome
Source: Philos Trans R Soc Lond B Biol Sci. 2014 Oct 5;369(1653):20130530. doi: 10.1098/rstb.2013.0530 (PMC4150305; doi:10.1098/rstb.2013.0530)

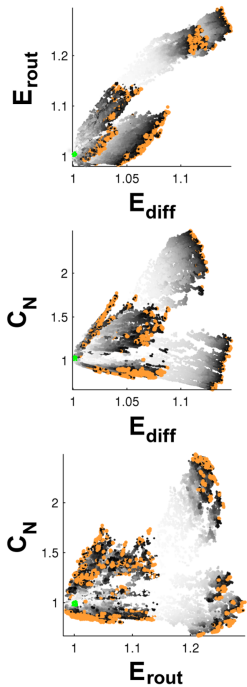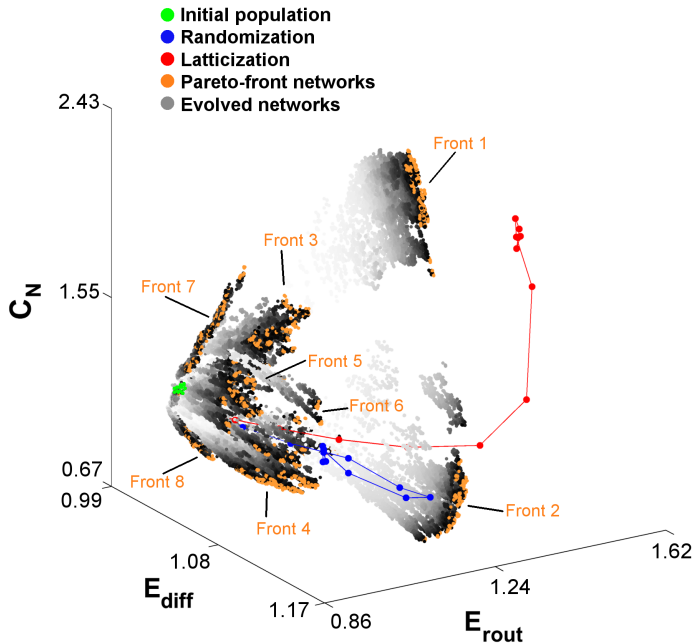

Supplement: Figure S1 [file rstb20130530supp1.pdf]

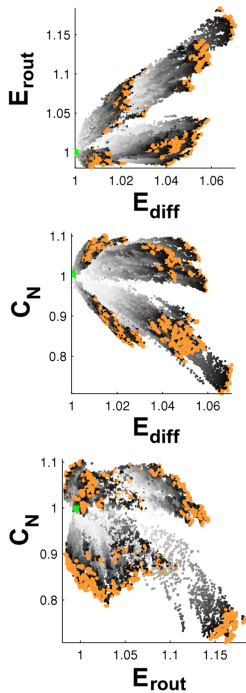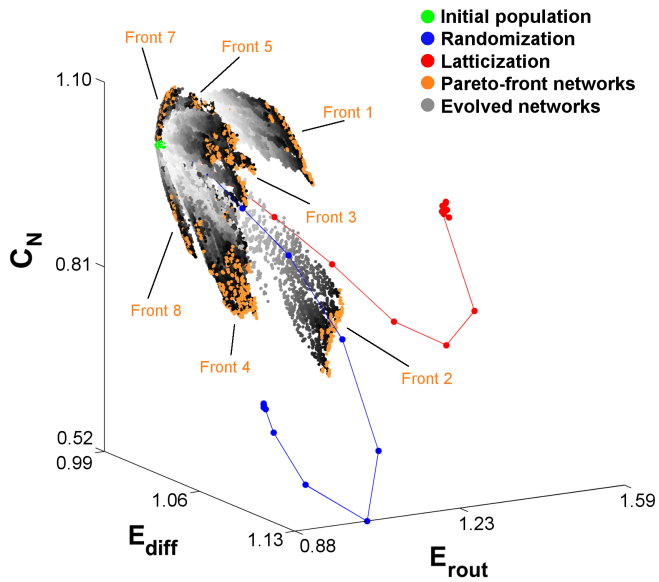

Supplement: Figure S2 [file rstb20130530supp2.pdf]
